# Community‐Engaged Course‐Based Undergraduate Research of Multidrug Resistance in *Escherichia coli* in Water Near Dairy and Hog Farms in Michigan

**DOI:** 10.1111/1758-2229.70151

**Published:** 2025-07-30

**Authors:** Renee Chowdhry, Soeun Jun, Yuwei Kong, Adrian Casillas Saenz, Jonathan Chung, Michelle Chang, Katie Osborn, Yuhui Zhang, Will Bodeau, Nicole Curristan, Brynn Sofro, Soham Ray, Karina Jimenez, Lynn Henning, Cole Dickerson, Salman Jaberi, Clare Delucchi, Jose Reyes Miranda, Adriane Jones, Carol Bascom‐Slack, Jennifer A. Jay

**Affiliations:** ^1^ Civil and Environmental Engineering UCLA Los Angeles California USA; ^2^ Department of Microbiology, Immunology, and Molecular Genetics UCLA Los Angeles California USA; ^3^ Socially Responsible Agriculture Project (SRAP) Salem Oregon USA; ^4^ Mount Saint Mary's University Los Angeles California USA; ^5^ Tufts University School of Medicine Boston Massachusetts USA

**Keywords:** antimicrobial resistance, concentrated animal feeding operations, ESBL‐EC, multidrug resistance

## Abstract

Antimicrobial resistance (AMR) was assessed in Michigan surface waters impacted by dairies, swine farms and human wastewater, as well as in an unimpacted (UI) comparison site. *Escherichia coli* (EC) was quantified in the presence and absence of cefotaxime, as extended‐spectrum beta‐lactamase‐producing EC (ESBL‐EC) has been deemed a proxy for AMR. Purified isolates of EC selected without antibiotics were characterised by disk diffusion; antibiotics tested included ampicillin, tetracycline, cefotaxime, cefoxitin, streptomycin, nalidixic acid, kanamycin, ciprofloxacin and erythromycin. Ampicillin and tetracycline resistance ranged up to 67% and 62% of the EC isolates, respectively, at livestock‐impacted sites, but were low at UI. Multidrug resistance (MDR) was not observed at all at UI but was observed in up to 76% and 67% of isolates from dairy and swine/dairy, respectively. AMP‐TE‐E was the most common resistance pattern observed, with all isolates originating from one of the dairy sites. Notably, resistance to cefotaxime did not correlate with MDR, indicating that preselection for ESBL‐EC before further AMR testing will not successfully characterise AMR or MDR from culturable EC. Interestingly, the percent of isolates resistant to AMP correlated quite well with MDR. This work highlights the importance of MDR characterisation at livestock‐impacted surface water sites.

## Graphical abstract

Through community‐engaged research, antimicrobial resistance was assessed in Michigan surface waters impacted by dairies, swine farms and human wastewater, as well as in an unimpacted comparison site. Multidrug resistance was observed at high levels from dairy and swine/dairy sites but not at all in unimpacted sites.

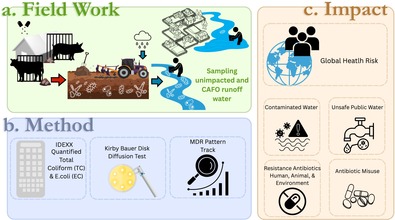

## 1 Introduction

Concentrated animal feeding operations (CAFOs) are known to be a source of environmental antimicrobial resistance (AMR) due to the use of antimicrobials in livestock production (Merchant and Martin 2024; Sayah et al. 2005). Offsite transport of antibiotic resistant bacteria (ARB) and antibiotic resistance genes (ARGs), including from land application of manure (He et al. 2020) and commercial garden products (Cira et al. 2021), can occur via air (Bai et al. 2022; McEachran et al. 2015; Sanchez et al. 2016), groundwater (Burch et al. 2023) and surface waters (Sayah et al. 2005). Environmental AMR is extremely complex to evaluate due to the plethora of possible microbial targets and antibiotic combinations and a current lack of standardised protocols. The World Health Organisation has deemed extended‐spectrum beta‐lactamase producing *Escherichia coli* (ESBL‐EC) to be one appropriate indicator of environmental AMR in the Tricycle Protocol (World Health Organization 2021), an accessible method for global monitoring of AMR. EC was chosen by the WHO due to its prevalence in the environment, ease of measurement and tendency to accumulate ARGs.

For assessment of AMR, this study focused on EC for several reasons. First, it is in accord with the WHO use of ESBL‐EC as a general indicator of AMR. Second, there are numerous papers in the literature that have characterised resistance patterns in EC with disk diffusion, so results can be compared to others. Third, EC has been shown to be elevated in streams near CAFOs (Lieberman and DuPont Golda 2023). Fourth, it is possible to modify commercially‐available kits with antibiotics in order to determine the fraction of total EC that can grow in the presence of antibiotics. Fifth, the methods for AMR EC by both disk diffusion of purified isolates and modification of IDEXX kits are feasible for undergraduates to learn and carry out within the time frame of one quarter.

CAFOs have been hypothesised to be a major source of multidrug resistance (MDR) due to the influence of both selective and co‐selective processes, as well as horizontal gene transfer. However, there are far fewer studies on MDR compared to AMR, particularly in the United States. Analysis of MDR is not straightforward, again due to the multiple possible targets and the variety of approaches (culture, qPCR and metagenomics). Whilst the Tricycle protocol includes culture‐based methods for further AMR testing of ESBL‐EC isolates, this method has been applied in many studies (Atlaw et al. 2022; Gao et al. 2015; Nossair et al. 2022; Nuangmek et al. 2018; Penati et al. 2024). It is critical to note that this preselection of ESBL‐EC from the general EC population does not allow for comparisons between impacted locations in the environment and nearby control sites, which may lack ESBL‐EC.

The method employed in this work involved EC‐selective plates without antibiotic additions followed by purification and testing for MDR. Thus, resistance profiles are reported for generic EC collected from impaired and less‐impacted sites. In this way, differences in MDR between impacted and unimpacted (UI) sites can be evaluated in terms of percent of isolates resistant to particular antibiotics, percent MDR and specific resistance patterns.

The laboratory work required to complete this project was largely conducted by undergraduate students taking part in a course‐based undergraduate research experience (CURE) for credit. CUREs allow all enrolled students to fully engage in authentic research without the barriers typically encountered by students wishing to be involved in undergraduate research (NASEM (National Academies of Sciences, Engineering, and Medicine) 2015). This project falls under the umbrella of the nationwide project Prevalence of Antimicrobial Resistance in the Environment (PARE), which seeks to fill research gaps in our understanding of environmental AMR by offering research training and engagement for undergraduates enrolled in CUREs (Fuhrmeister et al. 2021; Genné‐Bacon and Bascom‐Slack 2018; Genné‐Bacon et al. 2020). PARE and Tiny Earth are examples of successful CUREs that immerse students in authentic research experiences, functioning as effective educational tools and catalysts for advancing scientific knowledge (Hurley et al. 2021).

## 2 Materials and Methods

### 2.1 Community Engagement

This project was co‐created with Socially Responsible Agriculture Project (SRAP), a 501(c)(3) non‐profit organisation. For more than 20 years, SRAP has served as a mobilising force to help communities protect themselves from the damages caused by industrial livestock operations and to advocate for a food system built on regenerative practices, justice, democracy and resilience. Their team includes technical experts, independent family farmers and rural residents who have faced the threats of factory farms in their communities. When asked for help, SRAP offers free support, providing communities with the knowledge and skills to protect their right to clean water, air and soil and to a healthy, just and vibrant future. Project conception took place through a series of Zoom calls, and SRAP gave a tour of the sites for field collection.

### 2.2 Study Area

Study sites were chosen by SRAP in order to characterise AMR at sites near CAFOs with prior water quality violations as well as sites unimpacted by agriculture (one of which was impacted by wastewater). See Table 1 for site information. Sites D1 and D2 had dairy influence, whilst sites SD1–SD3 were in proximity to both swine and dairy CAFOs. The UI site was a pond not located directly near CAFOs. Whilst the WW site was also far from CAFOs, it did have some influence from human wastewater just upstream.

**TABLE 1:** Study sites.

Site	Type of livestock	Distance to nearest CAFO (m)	Size of nearest CAFO (head)	CAFOs within radius of 9 km	Total livestock in radius 9 km (head)
D1	Dairy cows	913	2400	4	8993
D2	Dairy cows	3740	2400	4	8993
SD1	Swine and dairy	1620	2350	2	6350
SD2	Swine and dairy	1439	3495	3	13,314
SD3	Swine and dairy	1070	6300	2	9795
UI	Unimpacted	11,910	1340	0	0
WW	Wastewater	83,180	2340	0	0

### 2.3 Sample Collection and Filtration

Water samples were collected between 6 and 9 am and were held on ice until same day processing in the laboratory. Total coliform (TC) and EC were quantified with Colilert‐18 (defined‐substrate technology developed by IDEXX). In addition to analysing samples according to manufacturer's instructions, samples were also analysed with added CTX (4 μg/mL) to quantify ESBL‐EC (Hornsby et al. 2023; Jimenez et al. 2025). To obtain EC for AMR testing by disk diffusion, water samples were filtered onto gridded 0.47 μm filters, which were cultured on mTEC plates with no amendments. After incubation for 2 h at 35°C and 22 h at 44.5°C, colonies were picked and streaked at least three times on TBX agar, with 24 h at 35°C incubation between restreaking. Purified cultures were preserved in 30%–50% glycerol and maintained at −60°C.

### 2.4 Kirby–Bauer Disk Diffusion Tests

The Kirby–Bauer disk diffusion test was used to determine the sensitivity and resistance of EC to nine antibiotics – cefotaxime (CTX/30 μg), tetracycline (TET/30 μg), ampicillin (AMP/10 μg), cefoxitin (FOX/30 μg), streptomycin (S/10 μg), nalidixic acid (NA/30 μg), kanamycin (K/30 μg), ciprofloxacin (CIP/5 μg) and erythromycin (E/15 μg).

Singular colonies were picked from the mTEC filtration plates and transferred to TBX plates using aseptic technique. Approximately 60–70 isolates were picked and continued for 3–4 purifications until it was clear a singular colony was left. These singular isolates were then transferred into MH (Mueller Hinton) Broth and were incubated to be used for AR testing. After the incubation period, 40 μL of the culture were pipetted onto MH plates and spread using a glass spreader. Nine different antibiotic disks were placed on to the plate and were incubated for 24 h at 35°C. The following day, the plates were taken out and the zone of inhibition was measured using a digital calliper for each of the nine antibiotics for each of the isolates and recorded. Zones were scored according to published values from CLSI (Clinical & Laboratory Standards Institute) (2023).

### 2.5 Secondary Confirmation Through Indole Testing

For secondary confirmation of EC, the indole test was conducted with the guidelines published by the American Society for Microbiology (MacWilliams 2009). The test was performed by adding 4 mL of tryptophan medium and EC from liquid cultures or frozen samples to a test tube. The test tubes were incubated for 24 h at 35°C. After incubation, five drops of Kovács reagents were added. A positive result, indicating the presence of EC, was observed by a red‐coloured ring, whilst a negative result had no change.

### 2.6 Course‐Based Undergraduate Research

Much of the laboratory work for this project was conducted by undergraduate students participating in a CURE. The CURE consisted of a two‐unit elective course led by a professor and one undergraduate tutor. The cap is set at eight students to ensure meticulous laboratory training and high quality results.

## 3 Results

Water quality was assessed for EC and TC both with the standard IDEXX protocol and with CTX amendment. For the unamended tests, TC ranged from 7270 to 19,863 MPN/100 mL at dairy sites and 4352–11,199 at swine and dairy‐impacted sites, whilst reaching only 798 MPN/100 mL at the UI site. The trend was similar for EC, which ranged from 61 to 365 MPN/100 mL at the dairy sites and 50–102 MPN/100 mL at the swine and dairy sites, whilst UI showed 15 and 3 MPN/100 mL for the two time points. The wastewater impacted site averaged 4283 and 79 MPP/100 mL for the two time points (Table 2).

**TABLE 2:** Faecal indicator bacteria results from the IDEXX procedure for surface waters near dairy and swine facilities as well as a nearby unimpacted site and a wastewater impacted site.

Type	April							May	
	D1	D2	SD1	SD2	SD3	UI	WW1	UI	WW2
TC (MPN/100 mL)	19,863	7270	11,199	15,531	4352	798	1296	816.4	7270
ESBL TC (MPN/100 mL)	345	1300	7215	411	83	88.8	345	12.1	59.8
TC (% resistance)	1.74	17.9	64.4	2.64	1.91	11.1	26.6	1.48	0.82
EC (MPN/100 mL)	61.3	365.4	74.3	50.4	102.2	15	51.2	3	107
ESBL‐EC (MPN/100 mL)	< 1	6.3	49.5	< 1	< 1	< 1	< 1	< 1	< 1
EC (% resistance)	0.00	1.72	66.62	0.00	0.00	0.00	0.00	0.00	0.00

*Note:* Sites D1 and D2 had dairy influence, whilst sites SD1–SD3 were in proximity to both swine and dairy CAFOs. The unimpacted (UI) and wastewater (WW) site were not located directly near CAFOs, although WW did have some influence from human wastewater.

At SD1, the fraction of TC resistant to CTX was 64%, which was much higher than that observed at all other sites (2%–11% at UI). For EC, the fraction of EC resistant to CTX was 67%, again higher than that seen at all other sites, even though the total EC measured by IDEXX was not especially high at SD1 (74 MPN/100 mL).

EC isolates from each site (total *n* = 202, between 8 and 48 per site) were purified and characterised for AMR (Table 3). TET resistance ranged from 0% to 62% at impacted sites (average of 23%), whilst the three sites not impacted by agriculture averaged 5% TET resistance. Similarly, AMP resistance ranged from 0% to 67% at agriculturally impacted sites (average of 27%), whilst less agriculturally impacted sites averaged 6% AMP resistance. Interestingly, the percent resistance to streptomycin was elevated only at SD1. FOX resistance was very low at all sites except for SD2, where 24% of isolates exhibited FOX resistance.

**TABLE 3:** Percent of EC isolates from each site that were resistant to the different antibiotics as assessed through Kirby–Bauer disk diffusion tests: cefotaxime (CTX/30 μg), tetracycline (TET/30 μg), ampicillin (AMP/10 μg), cefoxitin (FOX/30 μg), streptomycin (S/10 μg), nalidixic acid (NA/30 μg), kanamycin (K/30 μg), ciprofloxacin (CIP/5 μg), and erythromycin (E/15 μg).

Drug class	Antibiotics	D1 (*n* = 21)	D2 (*n* = 18)	SD1 (*n* = 20)	SD2 (*n* = 33)	SD3 (*n* = 8)	WW1 (*n* = 25)	WW2 (*n* = 48)	UI (*n* = 29)
Beta‐lactam antibiotics	CTX	0	16	0	0	0	0	0	0
	AMP	67	27	0	42	0	16	2	0
Tetracycline	TET	62	22	15	15	0	12	4	0
Cephalosporin antibiotics	FOX	5	0	0	24	0	0	2	0
Aminoglycoside antibiotics	S	14	33	60	33	12	40	15	17
	K	0	11	0	12	0	0	0	0
Quinolone antibiotics	NA	0	16	0	15	0	0	8	0
	CIP	0	16	0	18	0	0	0	0
Macrolide antibiotics	E	81	72	85	89	75	84	52	58

**FIGURE 1:**
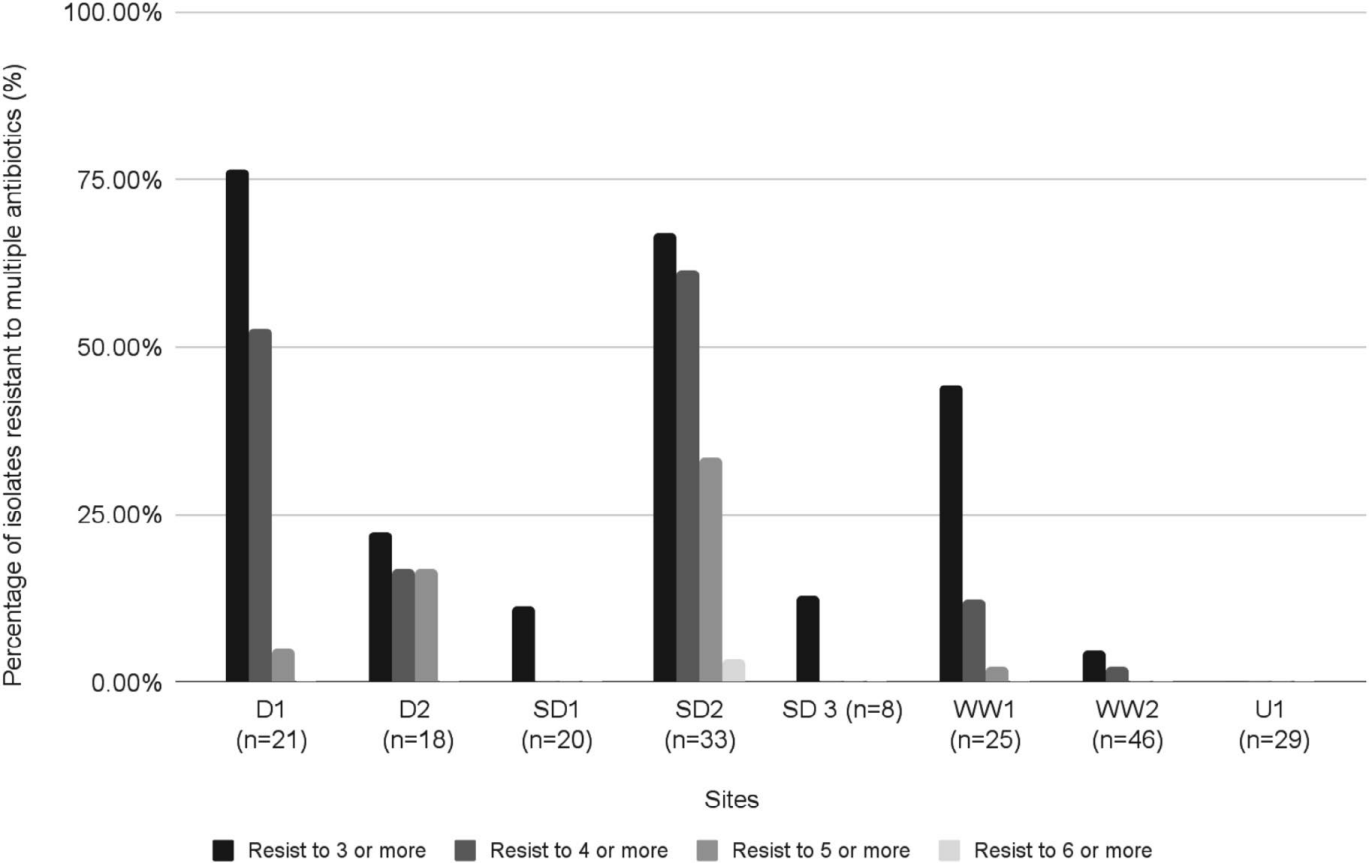
Percent of EC isolates showing MDR to three, four, five and six or more classes of antibiotics for each of the sites studied.

Differences between impacted and control sites were even more pronounced for MDR, with over 75% of EC isolates from one dairy site exhibiting resistance to three or more classes of antibiotics and over 50% of isolates resistant to four or more classes of antibiotics (Figure 1). Over 60% of isolates from a site impacted by both swine and dairy showed resistance to four or more antibiotics, and 30% of the isolates from the same site were resistant to five or more classes. Of the sites less impacted by agriculture, the site with some wastewater influence showed some MDR on one sampling occasion (approximately 44% of EC isolates resistant to three or more classes, and 12% resistant to four or more classes) but very little MDR on the second sampling. The site unimpacted by neither agriculture nor wastewater showed no MDR in any isolates.

The results showed that 30 isolates followed multi‐drug resistance that presented 15 different patterns (Table 4). AMP‐TE‐E was the most common pattern observed; all seven isolates with this pattern originated from site D1, which is the site with the highest levels of MDR. All three isolates resistant to five or more drug classes (AMP‐CTX‐S‐NA‐CIP‐E and AMP‐CTX‐TE‐S‐K‐NA‐CIP), and the one isolate resistant to six or more classes (AMP‐TE‐FOX‐S‐K‐NA‐CIP‐E), were from SD2; this site also showed the second highest levels of MDR.

**TABLE 4:** MDR patterns followed by isolates: cefotaxime (CTX/30 μg), tetracycline (TET/30 μg), ampicillin (AMP/10 μg), cefoxitin (FOX/30 μg), streptomycin (S/10 μg), nalidixic acid (NA/30 μg), kanamycin (K/30 μg), ciprofloxacin (CIP/5 μg) and erythromycin (E/15 μg).

MDR patterns	No. of drug class	Sites	No. of isolates	Percentage of isolates that follow the pattern (%)
AMP‐FOX‐S‐E	4	SD2	3	10.00
AMP‐S‐NA‐CIP‐E	4	SD2	2	6.67
AMP‐S‐NA‐E	4	SD2	1	3.33
FOX‐S‐E	3	SD2	1	3.33
S‐NA‐CIP‐E	3	SD2	1	3.33
AMP‐TE‐E	3	D1	7	23.33
AMP‐TE‐FOX‐S‐K	4	SD2	1	3.33
AMP‐TE‐FOX‐S‐K‐NA‐CIP‐E	6	SD2	1	3.33
AMP‐TE‐S	3	WW1	1	3.33
AMP‐TE‐S‐E	4	D1, WW1, UI	4	13.33
TE‐FOX‐E	3	SD1, UI	3	10.00
TE‐FOX‐S‐K‐E	4	SD2	1	3.33
TE‐S‐E	3	D2	1	3.33
AMP‐CTX‐S‐NA‐CIP‐E	5	D2	1	3.33
AMP‐CTX‐TE‐S‐K‐NA‐CIP	5	D2	2	6.67
Total			30	100.00

CTX resistance was only observed at one impacted site (16% of site 4 isolates), whilst resistance to other antibiotics and MDR was shown to be far more widespread (Table 3). So, there was not a correlation between percent CTX resistance in isolates (Figure S1). In addition, ESBL‐EC as measured with IDEXX also shows a weak correlation with MDR (Figures S2 and S3). IDEXX ESBL‐EC was highest (67% of total EC) at SD1 whilst MDR at this location was low.

**FIGURE 2:**
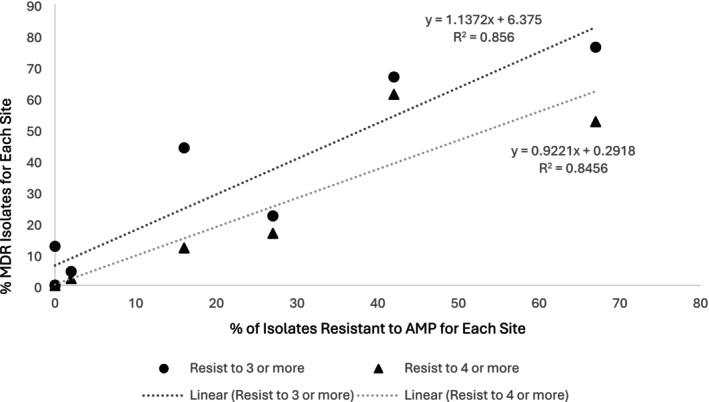
Percentage of isolates shown to be resistant to three and four or more classes of antibiotics versus percent resistant to ampicillin (AMP). Linear correlations are shown, with *p* = 0.0010 and *p* = 0.0012 for % of isolates resistant to three or more and four or more classes respectively.

Notably, the percent of isolates resistant to other antibiotics was a better predictor of MDR. Specifically, MDR to three or more and four or more classes of drugs was highly correlated to percent resistance to AMP, with *R* ^2^ of 0.86 (*p* = 0.0010) and 0.85 (*p* = 0.0012) respectively (Figure 2). TET also showed a linear correlation, with *R* ^2^ of 0.86 and 0.85 respectively for three or more and four or more classes of drugs (Figure S4). FOX showed a positive but non‐linear correlation (Figure S5).

## 4 Discussion

The findings here show highly elevated AMR and MDR in surface waters near dairy and swine facilities in Michigan compared to nearby comparison sites. AMP and TET, both of which are widely used in livestock production, showed higher average percent resistance for livestock‐impacted sites. Levels of TET resistance over 70% have been observed in EC from skin, faecal and wastewater samples from livestock facilities (Boripun et al. 2023; Fang et al. 2019).

The percent of isolates resistant to S and FOX was notably elevated at swine and dairy sites SD1 and SD2 respectively compared to dairy‐only sites. This may stem from the more prevalent use of antibiotics in swine compared to dairy production, where antibiotic use is most common in the calving stage. The percentage resistant to E was generally higher for agriculturally impacted sites, with levels ranging up to 81% and 89% for dairy and swine/dairy sites respectively whilst UI showed 58% resistance to E. This result is in accord with Mukuna et al. (2023), which showed all EC isolates from the cattle and goat farms they studied to be resistant to E as well as novobiocin.

Levels of TET and AMP resistance were generally elevated at livestock‐impacted sites, which is not surprising due to the use of tetracycline and beta‐lactam antibiotics in agriculture. The lack of beta‐lactam resistance observed in many of our isolates is in contrast to what was seen by Boripun et al. (2023) for pig faeces. The authors characterised AMR in 112 EC isolates from pig faeces and wastewater from pig farms in Southern Thailand. In that study, all of the isolates were resistant to beta‐lactam antibiotics.

The WHO's Tricycle Protocol currently recommends preselecting for ESBL‐EC from environmental samples and then further testing of the isolates for MDR. However, in this study, CTX resistance both shown in isolates and through the IDEXX testing was not a good predictor of general AMR or MDR in isolates. This is a critical finding, as the results here suggest that the methodology will not capture an accurate representation of the MDR present in the microbial population. Also, studying only ESBL‐EC from sites does not allow for a comprehensive comparison between impacted locations in the environment and nearby control sites, which may lack ESBL‐EC.

In this study, the percent of isolates resistant to AMP was highly correlated with MDR. However, this relationship might be site‐specific or industry‐specific. King and Schmidt (2017) screened 150 EC isolates from South African herbivores and domestic pigs, and only 2 of the 19 MDR EC isolates were resistant to AMP. None of the 28 EC isolated from bioreactors at pig farms in Brazil were resistant to AMP (Oliveira‐Silva et al. 2024). In a study conducted in Michigan farms (Sayah et al. 2005), 5.29% of all isolates (*n* = 2552) were resistant to AMP. Most MDR isolates were resistant to TET and another antibiotic. MDR with AMP resistance was detected in 24.5% of the MDR isolates.

In this study, percent MDR was over 50% at one of the swine sites and over 75% at one of the dairy sites. The values are in the range of what has been seen elsewhere at farm settings. In the Boripun et al. (2023) study of pig faeces and wastewater, MDR was prevalent (but not at levels as high as the most elevated levels seen in this study), with 48.2% of isolates (54/112) exhibiting resistance to three or more classes of antibiotics. There were 11 distinct patterns, with 27.7% of total isolates resistant to three classes, 17.8% resistant to four classes and 2.68% (3/112) resistant to five classes. Fang et al. (2019) also investigated AMR in EC strains from a large‐scale swine slaughterhouse and its downstream markets in Zhejiang, China. Three hundred isolates were identified from 720 samples; isolates were all collected from lairages and downstream open markets. Isolates showed the highest resistance to TET, and 80.67% of the *E. coli* isolates were identified as MDR. Shoaib et al. (2023) investigated AMR in EC strains from a dairy farm in Xinjiang, China. Of the 338 strains, 84% were resistant to at least one antibiotic, and of these, 44% of the resistant strains were identified as MDR. Most of the strains (63.43%) were resistant to trimethoprim/sulfamethoxazole, followed by cefotaxime (44.08%), ampicillin (33.73%), ciprofloxacin (31.36%), tetracycline (28.99%), and less to florfenicol (7.99), gentamicin (4.44%), amikacin (1.77%) and fosfomycin (1.18%).

Moreover, Shoaib et al. (2023) investigated AMR in EC strains from a dairy farm in Xinjiang, China. They collected 209 samples from manure slurry, faecal matter, raw milk, crop soil and blank soil. From these samples, 338 strains were confirmed by 16s rRNA gene amplification and 84% were resistant to at least one antibiotic. In a recent study at cattle and goat farms in Tennessee, all Enterobacter (including EC) showed 100% resistance to novobiocin, erythromycin and vancomycin (EC is intrinsically resistant to vancomycin, so this should not be counted for MDR determination). There were three different resistance patterns for EC; NOV‐ERY‐VAN was observed at both cattle farms and the goat farm. NOV‐ERY‐TET‐VAN was the most prevalent at all three sites. NOV‐TET‐ERY‐VAN‐KAN was seen only at the goat farm (Mukuna et al. 2023). Also, Brisola et al. (2019) assessed AMR in EC from 100 pig farms in Southern Brazil, finding 37% of the isolates were resistant to three or more classes of antibiotics.

Whilst the studies discussed above present MDR results for farm environments, there are far fewer studies of AMR and MDR in EC in agriculturally impacted surface waters, particularly in the United States. In one study from 2005, EC isolates were obtained from livestock faeces, the farm environment, human septage and local surface waters in Michigan (Sayah et al. 2005). Whilst AMR to all antibiotics tested and MDR were observed in isolates from the first three types of samples, all of the isolates from surface waters were susceptible to all antibiotics tested other than cephalothin. Results from this study are the first to our knowledge showing elevated MDR in EC in surface waters downstream of CAFOs in the United States. The findings point to a need for greater surveillance and provide necessary information on potential methods.

This work provides a successful example of a CURE applied to expand our knowledge of environmental AMR. CUREs are known to be an inclusive method of teaching (Bangera and Brownell 2014), addressing the current underrepresentation of women and underrepresented minorities (URMs) in the science, technology, engineering and maths (STEM) fields (Callejas et al. 2023; Werth et al. 2022). The methods employed here are exceptionally well‐suited to CUREs in microbiology and environmental science, as students are able to participate in all aspects of research including sample collection, isolate purification, secondary confirmation, AMR testing and data analysis within one quarter or semester even without prior laboratory experience. Whilst AMR is known to be prevalent at CAFOs, the level of off‐site transport and the extent of MDR are currently understudied. More CUREs in this area will help address these gaps.

## Author Contributions

**Renee Chowdhry:** investigation, data curation, formal analysis, conceptualization, methodology, project administration, supervision, validation, visualization writing – original draft, writing – review and editing. **Soeun Jun:** investigation, data curation, formal analysis, conceptualization, methodology, project administration, supervision, validation, visualization writing – original draft, writing – review and editing. **Yuwei Kong:** investigation, conceptualization, methodology, writing – review and editing, data curation, supervision, formal analysis, project administration. **Adrian Casillas Saenz:** investigation, writing – review and editing, data curation, formal analysis. **Jonathan Chung:** investigation, data curation, writing – review and editing. **Michelle Chang:** investigation, writing – review and editing, data curation, formal analysis, visualization. **Katie Osborn:** investigation, conceptualization, methodology, writing – review and editing, data curation, supervision, formal analysis. **Yuhui Zhang:** investigation, conceptualization, methodology, writing – review and editing, data curation, supervision, formal analysis. **Will Bodeau:** investigation, data curation, writing – review and editing. **Nicole Curristan:** investigation, data curation, supervision, writing – review and editing. **Brynn Sofro:** investigation, writing – review and editing. **Soham Ray:** investigation, writing – review and editing. **Karina Jimenez:** investigation, conceptualization, methodology, writing – review and editing, data curation, supervision, formal analysis. **Lynn Henning:** conceptualization, investigation, writing – review and editing. **Cole Dickerson:** conceptualization, investigation, writing – review and editing. **Salman Jaberi:** conceptualization, investigation, writing – review and editing. **Clare Delucchi:** writing – review and editing, visualization, methodology. **Jose Reyes Miranda:** writing – review and editing, visualization. **Adriane Jones:** conceptualization, data curation, formal analysis, writing – review and editing. **Carol Bascom‐Slack:** conceptualization, investigation, writing – review and editing. **Jennifer A. Jay:** investigation, data curation, formal analysis, conceptualization, methodology, project administration, supervision, validation, visualization writing – original draft, writing – review and editing, fund management, resources.

## Conflicts of Interest

The authors declare no conflicts of interest.

## Supporting information


**Data S1.** Supplementary information.

## Data Availability

The data that support the findings of this study are available on request from the corresponding author. The data are not publicly available due to privacy or ethical restrictions.
