# Supplementary material for: Community‐Engaged Course‐Based Undergraduate Research of Multidrug Resistance in Escherichia coli in Water Near Dairy and Hog Farms in Michigan
Source: Environ Microbiol Rep. 2025 Jul 30;17(4):e70151. doi: 10.1111/1758-2229.70151 (PMC12309979; doi:10.1111/1758-2229.70151)
Supplement: Supplementary file 1 — Data S1. Supplementary information. [file EMI4-17-e70151-s001.pdf]

SUPPLEMENTAL INFORMATION FOR

Community-engaged course-based undergraduate research of multidrug resistance in *Escherichia coli* in water near dairy and hog farms in Michigan

Renee Chowdhry[1,2], Soeun Jun[1], Yuwei Kong[1], Adrian Casillas[1], Jonathan Chung[1], Michelle Chang[1], Katie Osborn[1], Yuhui Zhang[1], Brynn Sofro[1], Will Bodeau[1], Soham Ray[1], Karina Jimenez[1], Lynn Henning[2], Cole Dickerson[2], Salman Jaberi[1], Clare Delucchi[1], Jose Reyes Miranda[1], Adriane Jones[3], Carol Bascom-Slack[8], Jennifer A. Jay[3]

[1] Civil and Environmental Engineering, UCLA, Los Angeles, CA, USA
[2] Dept of Microbiology, Immunology, and Molecular Genetics, UCLA, Los Angeles, CA, USA
[3] Socially Responsible Agriculture Project (SRAP), USA
[4] Mount Saint Mary's University, Los Angeles, CA, USA
[5] Tufts University, Medford, MA, USA

Item	Page
Figure S1. Percent of MDR EC isolates versus the percent CTX resistant.	2
Figure S2. Percent of MDR EC isolates versus the percent TET resistant.	2
Figure S3. Percent of MDR EC isolates versus the percent FOX resistant.	3
Figure S4. Percent of MDR EC isolates versus the total amount of ESBL-EC.	3
Figure S5. Percent of MDR EC isolates versus the percent of ESBL-EC resistant.	4

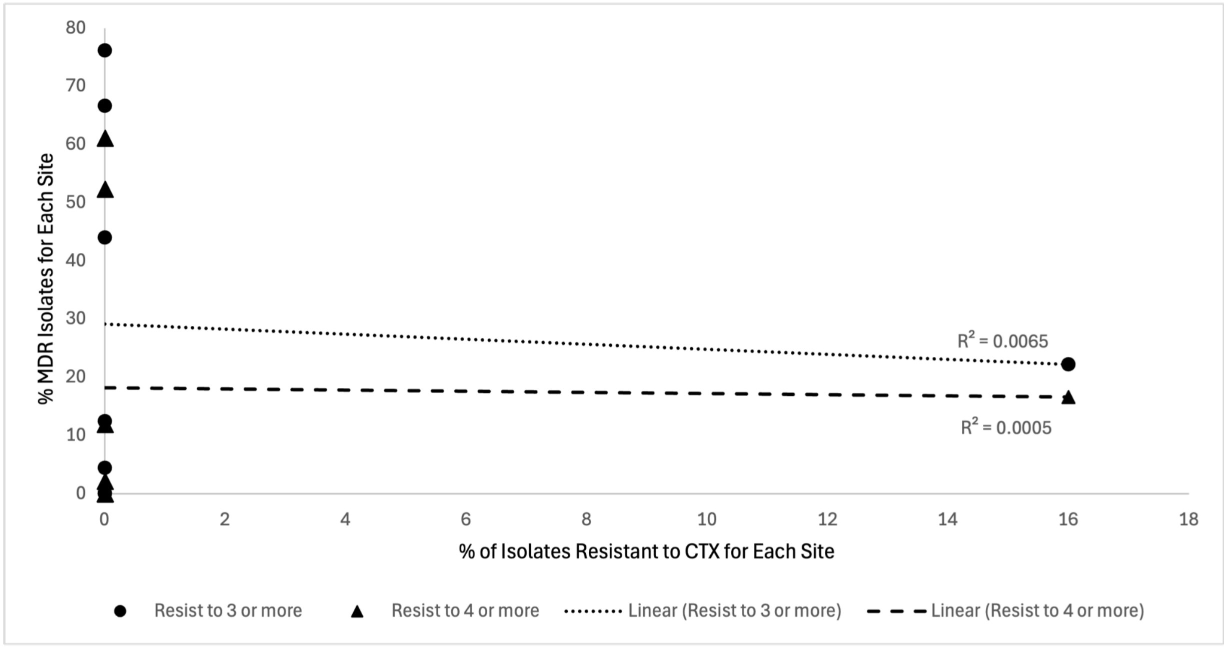

Figure S1. Percent of MDR EC isolates versus the percent cefotaxime (CTX) resistant. For linear correlations, p values are 0.85 and 0.96 for % of isolates resistant to 3 or more and 4 or more classes, respectively.

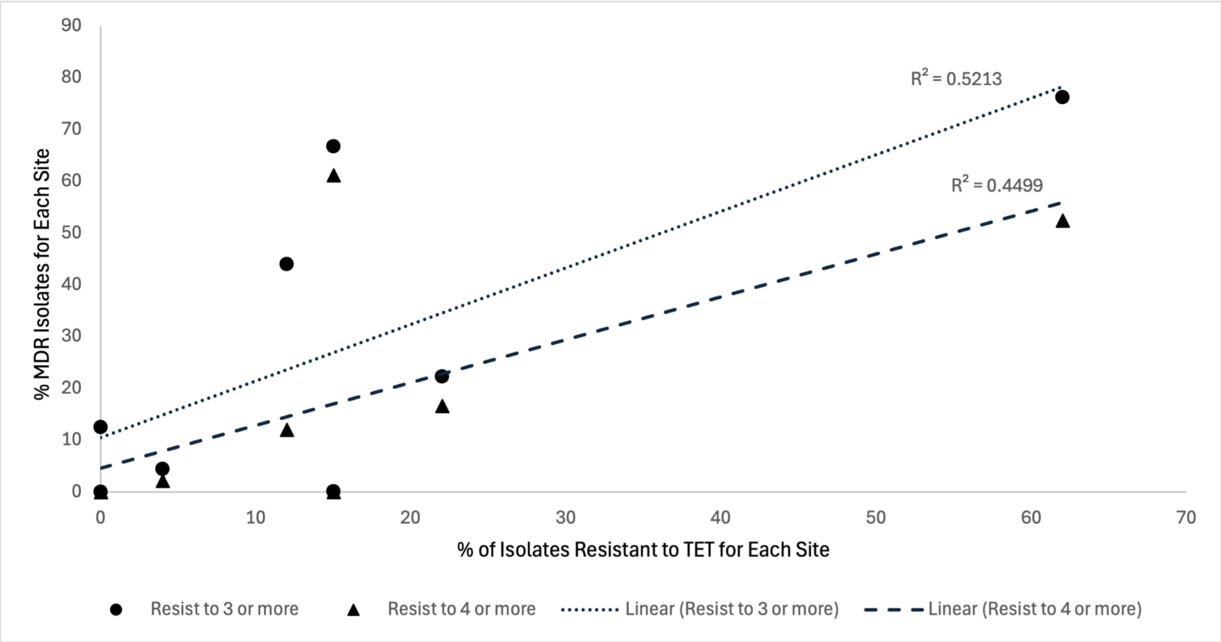

Figure S2. Percent of MDR EC isolates versus the percent tetracycline (TET) resistant. For linear correlations, p values are 0.043 and 0.069 for % of isolates resistant to 3 or more and 4 or more classes, respectively.

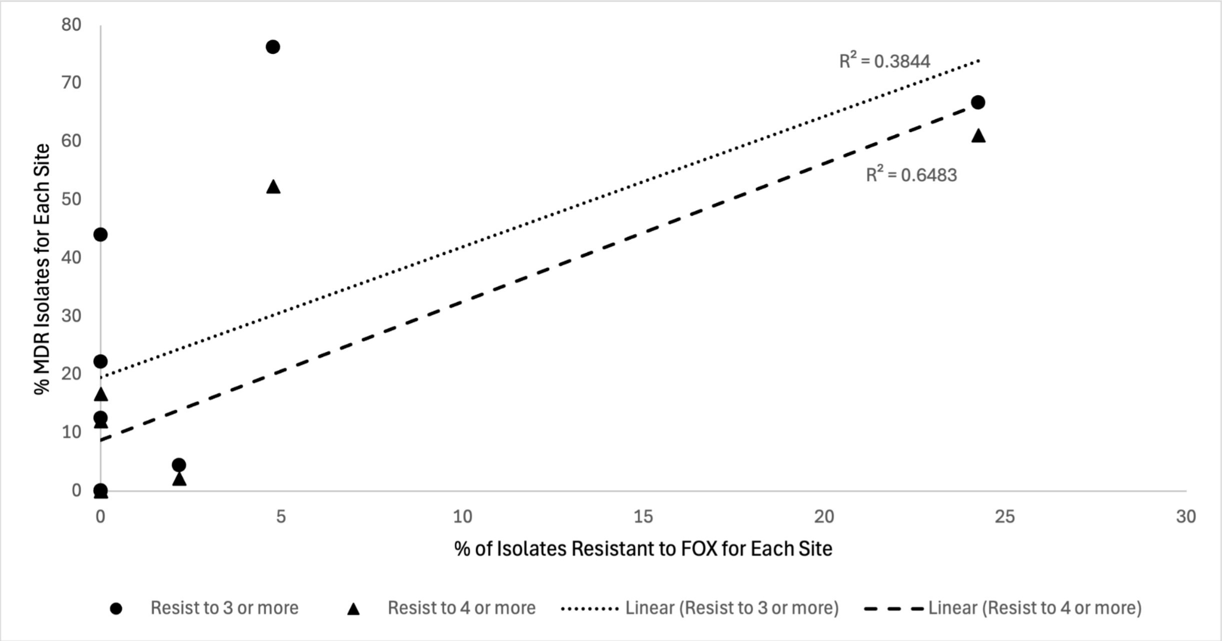

Figure S3. Percent of MDR EC isolates versus the percent cefoxitin (FOX) resistant. For linear correlations, p values are 0.10 and 0.0.016 for % of isolates resistant to 3 or more and 4 or more classes, respectively.

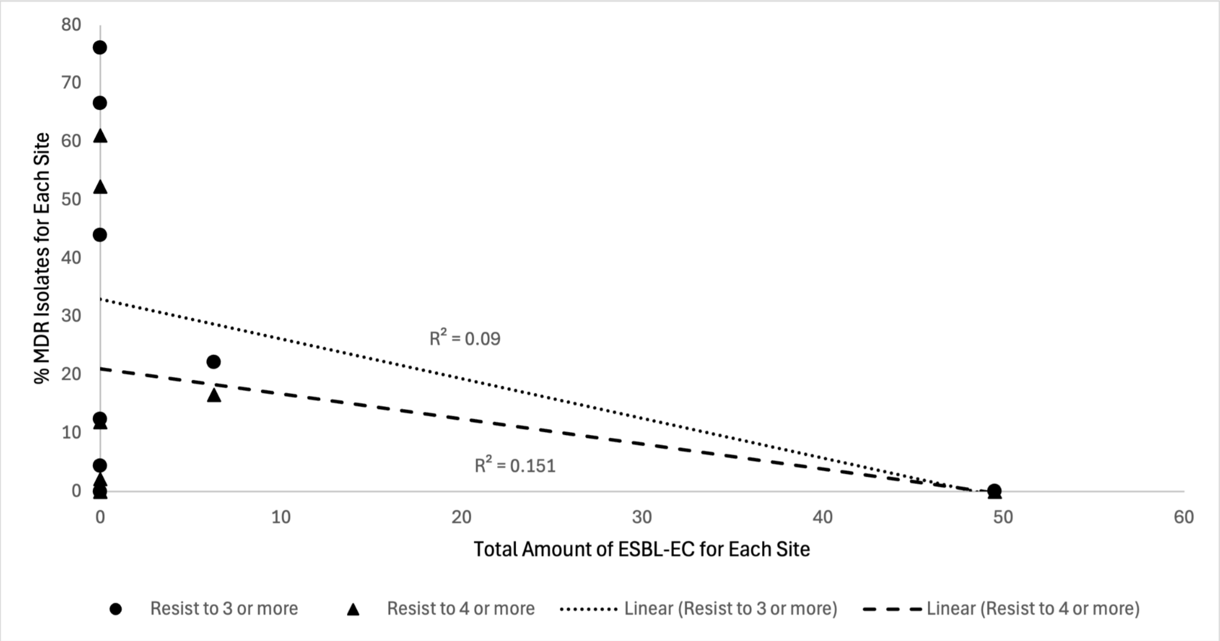

Figure S4. Percent of MDR EC isolates versus the total amount of ESBL-EC. For linear correlations, p values are 0.34 and 0.47 for concentration of ESBL-EC resistant to 3 or more and 4 or more classes, respectively.

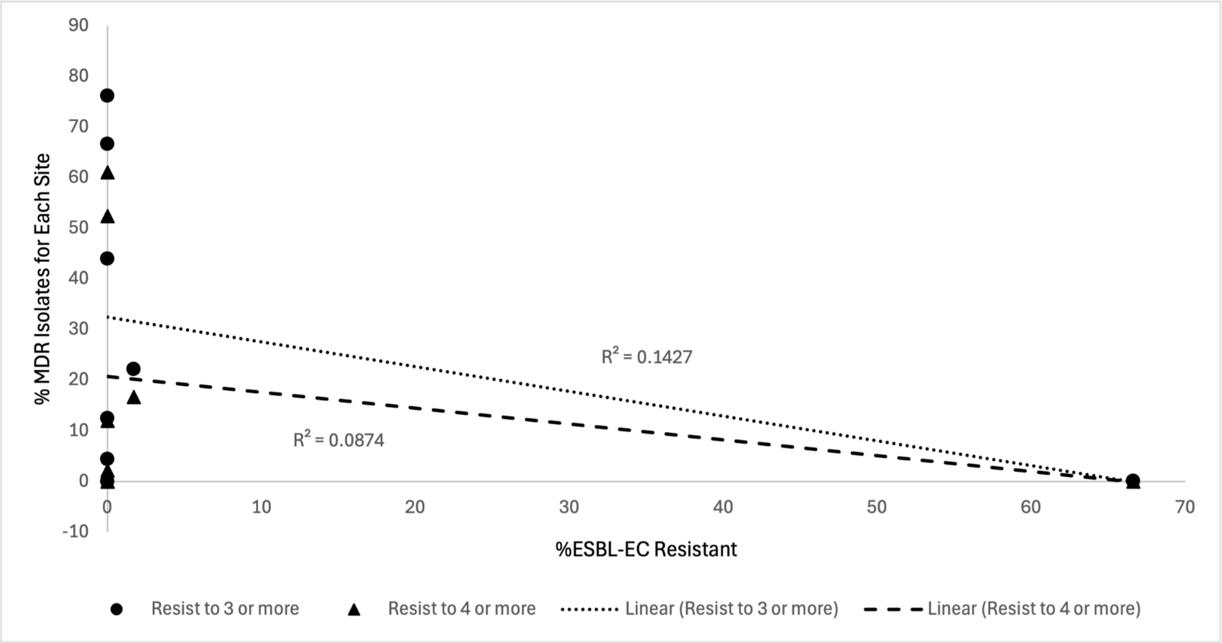

Figure S5. Percent of MDR EC isolates versus the percent of ESBL-EC resistant. For linear correlations, p values are 0.36 and 0.48 for % of ESBL-EC resistant to 3 or more and 4 or more classes, respectively.
